# ﻿Rediscovery of *Histiotusalienus* Thomas, 1916 a century after its description (Chiroptera, Vespertilionidae): distribution extension and redescription

**DOI:** 10.3897/zookeys.1174.108553

**Published:** 2023-08-14

**Authors:** Vinícius C. Cláudio, Brunna Almeida, Roberto L. M. Novaes, Marcos A. Navarro, Liliani M. Tiepolo, Ricardo Moratelli

**Affiliations:** 1 Fundação Oswaldo Cruz, Fiocruz Mata Atlântica, Rio de Janeiro, RJ, Brazil Fundação Oswaldo Cruz, Fiocruz Mata Atlântica Rio de Janeiro Brazil; 2 Universidade Federal do Paraná, Setor Litoral, Matinhos, PR, Brazil Universidade Federal do Paraná Matinhos Brazil

**Keywords:** Bats, diagnosis, morphology, Neotropics, taxonomy

## Abstract

*Histiotus* is a Neotropical genus of bat that currently includes 11 species. The systematics of *Histiotus* has been the focus of several studies over the last decades. However, no broad systematic revision has been made, and taxonomic issues such as synonymies, use of subspecies, and specimens that do not fit the description of valid species still persist, as pointed out by several authors. *Histiotusalienus* was described in 1916 and is known only by the holotype. Here we present a second record of *H.alienus* and an amended diagnosis of this species. We use qualitative, quantitative, and morphometric analyses based on data from 184 specimens of *Histiotus* and almost all valid species. Our amended diagnosis establishes the taxonomic limits of *H.alienus*, as well as a comprehensive comparison with congeners. We also explore new diagnostic characters for *H.alienus* and provide a few notes on the natural history of this species. Our results highlight skull similarities among *Histiotus* species and reinforce the usefulness of external morphology for their correct identification. Despite our new insights into the taxonomy of the genus, several taxonomic issues remain, and a comprehensive revision of the genus is needed.

## ﻿Introduction

*Histiotus* Gervais, 1856 is endemic to South America and currently includes 11 species (Cláudio 2019; Velazco et al. 2021). The number of species recognized in the genus, however, has varied over the last decades (see Simmons 2005; Handley and Gardner 2008; Cláudio 2019; Rodríguez-Posada et al. 2021), and three new species were recently described (Feijó et al. 2015; Rodríguez-Posada et al. 2021; Velazco et al. 2021). The generic status of *Histiotus* has also been debated over the last decades since the genus is consistently recovered within *Eptesicus* in molecular phylogenies (= *Eptesicus* Rafinesque, 1820 + *Cnephaeus* Kaup, 1829 + *Neoeptesicus*Cláudio et al., 2023; see Cláudio et al. 2023). *Eptesicus* (*sensu lato*) occurs in both the New and Old World (Hoofer and Van Den Bussche 2003; Roehrs et al. 2010, 2011; Juste et al. 2013; Yi and Latch 2022). To solve the paraphyly of *Eptesicus* (*sensu lato*), two differing arrangements were proposed and have been used since: (1) to treat *Histiotus* as a subgenus of *Eptesicus* (*sensu lato*); or (2) to treat *Histiotus* as a full genus and allocate Old World forms of *Eptesicus* (*sensu lato*) in the available name *Cnephaeus* (Hoofer and Van Den Bussche 2003; Roehrs et al. 2010, 2011; Juste et al. 2013; Yi and Latch 2022). More recently, Cláudio et al. (2023) reevaluated the taxonomy of New World *Eptesicus* (*sensu lato*) and *Histiotus* and proposed a new arrangement based on molecular and morphological data. In this arrangement, *Histiotus* is treated as a full genus, while *Eptesicus* (*sensu lato*) was split into three separate genera: *Eptesicus*, which includes only *E.fuscus* and *E.guadeloupensis*; *Cnephaeus*, which includes all Old World species of the former *Eptesicus*; and *Neoeptesicus*, a newly described genus which includes the Neotropical and smaller species of *Eptesicus* (*sensu lato*; Cláudio et al. 2023). Here we follow Cláudio et al. (2023) in treating *Histiotus* at the genus level.

Despite the continued efforts on the taxonomy of *Histiotus*, no broad revision has been hitherto made, and several problems are still persist in the systematics of the genus, such as the use of name combinations, subspecies and synonyms, specimens that do not fit the species description, and species that are poorly known (Handley and Gardner 2008; Cláudio 2019; Rodríguez-Posada et al. 2021; Velazco et al. 2021). *Histiotusalienus* was described by Oldfield Thomas in 1916, and only its holotype, captured in Joinvile, Santa Catarina state, southern Brazil, has been known ever since. Most authors consider this species valid, but it has also been treated either as a subspecies of *H.montanus* or as part of *H.macrotus*, and its taxonomic status is still uncertain (Handley and Gardner 2008; Cláudio 2019). Except for the brief morphological description and little more than a dozen measurements available in the original description of *H.alienus*, knowledge of this species is limited, and a fuller diagnosis and comparisons with its congeners is imperative.

Here, we present the second known record of *H.alienus*, a specimen captured by us in Paraná state in southern Brazil. In addition, we also provide an amended diagnosis of *H.alienus* based on our morphological analysis of the two known specimens, and we offer a detailed comparison with all congeners.

## ﻿Methods

The second specimen of *Histiotusalienus* was captured during a field survey in November 2018 in the Refúgio de Vida Silvestre dos Campos de Palmas (Palmas Grasslands REVIS), which is a 16,600-ha protected area in southern Brazil. Palmas Grasslands REVIS encompasses mainly natural grasslands with small, isolated fragments of moist *Araucaria* forest and anthropized areas that include agriculture and silviculture patches (ICMBio 2014). The surrounding region of the reserve has wind farms for energy generation, which negatively impact the bat fauna. Bats were sampled using 10 mist nets (9 × 3 m, 20 mm mesh) placed at forest edges, on trails across forest patches, over water bodies, and near previously identified roosts. Mist nets remained open for 6 h each night from sunset and were inspected at average intervals of 30 min. All field activities followed biosafety and bioethics standards and have legal permission (SISBIO 19037-1; SISBIO 63846-1; CEUA-Fiocruz LM-6/18; SisGen A0E5902).

For taxonomic comparisons and species redescription, we analyzed 184 specimens of *Histiotus* (Appendix 1), including almost every species of the genus except for *Histiotuscadenai*Rodríguez-Posada et al. 2021. Specimens are deposited in the following zoological collections: Coleção Adriano Lúcio Peracchi, Instituto de Biologia, Universidade Federal Rural do Rio de Janeiro, Seropédica, Brazil (**ALP**), American Museum of Natural History, New York, USA (**AMNH**), Natural History Museum, London, United Kingdom (**BM**), Colección Mamíferos Lillo, Tucumán, Argentina (**CML**), Field Museum of Natural History, Chicago, USA (**FMNH**), Louisiana State University, Museum of Natural Science, Baton Rouge, USA (**LSU**), Museo Argentino de Ciencias Naturales, Buenos Aires, Argentina (**MACN**), Museu Nacional da Universidade Federal do Rio de Janeiro, Rio de Janeiro, Brazil (**MN**), Muséum national d’Histoire naturelle, Paris, France (**MNHN-ZM-MO**), and National Museum of Natural History, Washington, D.C., USA (**USNM**).

For qualitative, quantitative, and morphometric analyses, we used external (*N* = 3) and craniodental (*N* = 16) measurements based on, but not limited to, Thomas (1916), Handley and Gardner (2008), Feijó et al. (2015), Rodríguez-Posada et al. (2021), and Velazco et al. (2021). Both external and skull measurements are presented and described in Table 1 and were taken from adults with closed epiphyses. All the measurements were taken with digital calipers, and craniodental measurements were determined under binocular microscopes with low magnification (usually 6×) to the nearest 0.01 mm. Color nomenclature used in the description of the species follows Ridgway (1912).

**Table 1.** External and craniodental measurements taken from *Histiotus* specimens.

Abbreviation	Measurement	Description
FL	Forearm length	Elbow to distal end of forearm including carpals
EL	Ear length	Ear notch to tip of pinna
WMLE	Width of medial lobe of ear	Maximum width of medial lobe of pinna
MAL	Mandibular length	Canine to the condyloid process
MAN	Mandibular toothrow length	From the lower canine to third molar
COH	Height of coronoid process	Perpendicular height from tip of coronoid process to base of mandible
GLS	Greatest length of skull, including incisors	Apex of upper internal incisors to occiput
CCL	Condylo-canine length	Anterior surface of upper canines to a line connecting occipital condyles
CIL	Condylo-incisive length	Apex of upper internal incisors to a line connecting occipital condyles
BAL	Basal length	Least distance from apex of upper internal incisors to anterior margin of foramen magnum
ZYG	Zygomatic breadth	Greatest breadth across outer margins of zygomatic arches
MAB	Mastoid breadth	Greatest breadth across mastoid region
BCB	Braincase breadth	Greatest breadth of globular part of braincase
POB	Postorbital constriction	Least breadth across frontals posterior to postorbital bulges
BAC	Breadth across canines	Greatest breadth across outer edges of crowns of upper canines including cingulae
BAM	Breadth across molars	Greatest breadth across outer edges of crowns of upper molars
MTL	Maxillary toothrow length	Upper canine to third molar
M1M3	Upper molar toothrow length	M1 to M3
WFH	Width of foramen magnum	Greatest width between internal margins of foramen magnum, in a horizontal axis

For morphometric analyses, we employed a canonical variate analysis (CVA) using all 16 craniodental measurements. The CVA was used to discriminate samples and compare skull morphology among *Histiotus* species. The analysis was performed in R (R Development Core Team 2020), using the MASS (Venables and Ripley 2002) and Lattice (Sarkar 2008) packages. To balance the number of samples within each species, we selected a subset of the total specimens analyzed, including a total of 58 samples, as follows: *H.alienus* (*N* = 2), *H.colombiae* (*N* = 6), *H.humboldti* (*N* = 4), *H.laephotis* (*N* = 10), *H.macrotus* (*N* = 8), *H.magellanicus* (*N* = 10), *H.montanus* (*N* = 8), and *H.velatus* (*N* = 10). Specimens with incomplete datasets were removed from the analysis, and eventual missing values (<5% of the total dataset) were estimated from the existing raw data using the Amelia II package (Honaker et al. 2011) implemented in R.

## ﻿Results

**Figure 1.**
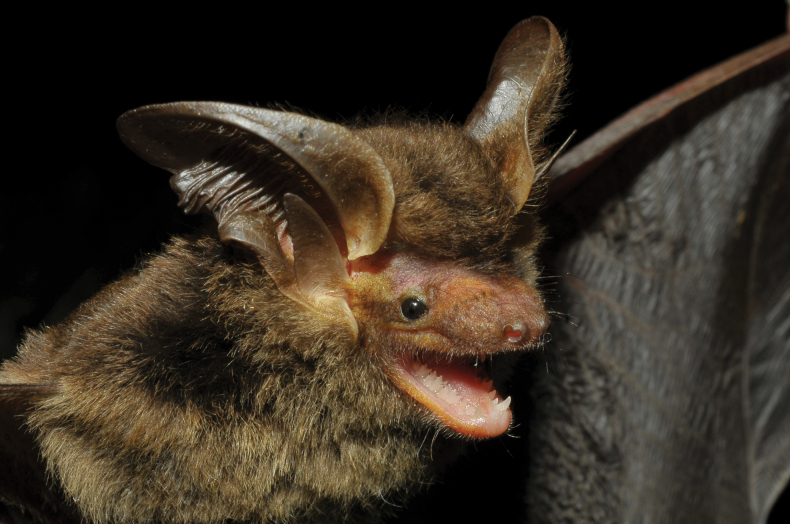
Adult male of *Histiotusalienus* (MN 91624), captured on the municipality of Palmas, Paraná state, Brazil.

**Figure 2.**
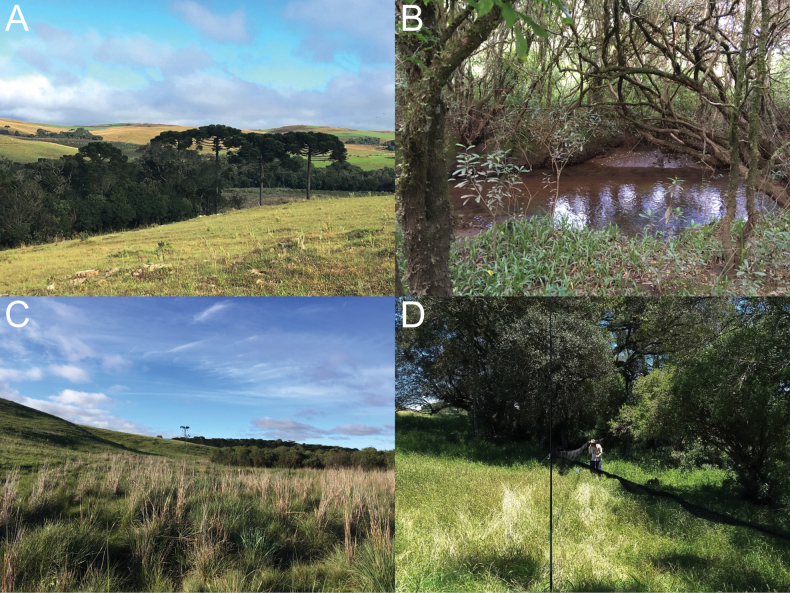
Cerro Chato Farm, Palmas Grasslands REVIS **A** general aspect of the Palmas Grasslands REVIS **B** interior of the riparian forest along Chopim River **C** aspect of the natural grassland and Araucaria forests vegetation **D** mist nets set at the edge of a forest fragment where the second specimen of *Histiotusalienus* was captured. Photos: **A–C** Liliani M. Tiepolo, 2018 **D** Marcos Navarro, 2018.

**Figure 3.**
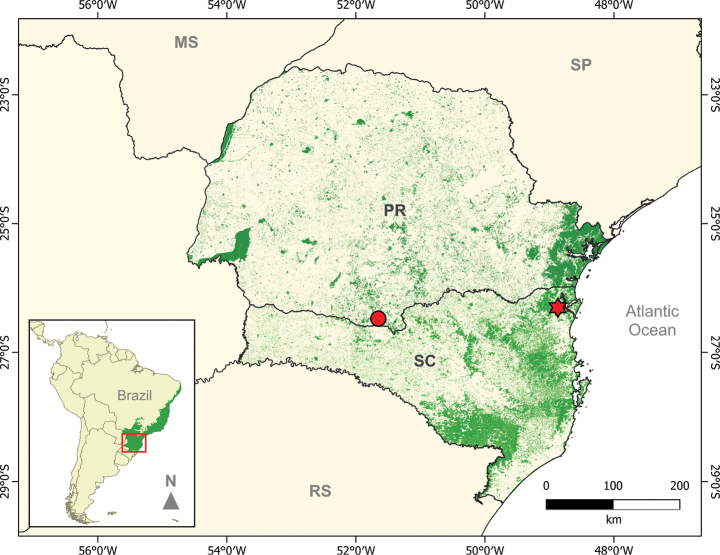
Known localities of *Histiotusalienus* in southern Brazil: type locality in Joinville, Santa Catarina, SC (red star); new record (MN 91624) in the municipality of Palmas, Paraná, PR (circle). The green areas represent remnants of Atlantic Forest.

On 21 November 2018, we captured an adult male of *H.alienus* (Fig. 1) in a mist net set at ground level 4h after sunset (ca 11 pm). It had been foraging on the edge of a forest fragment next to a grassland. The area is located in Cerro Chato Farm (26°30'10"S, 51°40'04"W, 1208 m a.s.l.; Figs 2, 3), within the Chopim River hydrographic basin. This river is one of the main tributaries of Iguaçu River in the Palmas Plateau, a subdivision of the Brazilian Southern Plateau. It was collected and preserved in spirit (alcohol 70 °GL) with the skull removed and deposited in Museu Nacional da Universidade Federal do Rio de Janeiro (MN 91624). At the same site, we also collected the following vespertilionid species: *Myotisriparius* Handley, 1960, *Myotisruber* (É. Geoffroy, 1806), *Neoeptesicusfurinalis* (d’Orbigny & Gervais, 1847), and *Neoeptesicustaddeii* (Miranda, Bernardi & Passos, 2006).

The only known record of *H.alienus* until now was from the type locality in Joinville, Santa Catarina state, southern Brazil. With the new record from Palmas, Paraná state, we expand the geographic distribution of this species by about 280 km to the west at the same latitude (Fig. 3). The new record is from *Araucaria* forest, a different type of forest cover than at the type locality. *Araucaria* forests are even more threatened than the coastal Atlantic Forest. Thus, *H.alienus*, is now considered to occur from dense rainforests to *Araucaria* and riparian forests and grasslands, at altitudes from sea level to over 1200 m a.s.l.

### ﻿Systematics

**Genus *Histiotus* Gervais, 1856**

#### ﻿ Histiotus alienus

Taxon classificationAnimaliaChiropteraVespertilionidae

Thomas, 1916

374D8EF0-3917-5C94-BC32-BACB40785D27

##### Materials examined.

***Holotype*.** Brazil • 1 ♀; Santa Catarina state, Joinville; sea level; W. Ehrhardt leg.; BM 9.11.19.1. **Other specimens.** Brazil • 1 ♂; Paraná state, Palmas; 26°30'10"S, 51°40'04"W; 1208 m a.s.l.; 21 Nov. 2018; Vinícius C. Cláudio and Marcos A. Navarro leg.; 21 Nov. 2018; in mist-net; MN 91624.

##### Distribution.

*Histiotusalienus* is known only from two localities in southern Brazil, one each in Santa Catarina (Joinville) and Paraná (Palmas) states.

##### Diagnosis.

*Histiotusalienus* is distinguished from all other congeners by the following combination of characters: bicolored and dark dorsal fur; ventral fur bicolored and only slightly lighter than dorsal fur; ears intermediate in size when compared to congeners (EL ~ 27.5 mm) and slightly triangular; medial lobe of ear small (WMLE ~ 4.5 mm); transverse band of skin between pinnae low, 1–2 mm high at the edges and weakly fading toward the central portion, where it is practically absent.

##### Description.

*Histiotusalienus* is a medium-sized species within the genus (FL 43.3–44.5 mm; Table 2). Dorsal fur long (LDF ~ 11.5 mm) and bicolored, with Bone Brown bases that extend to about half the length of hairs and Light Brownish Olive tips; contrast between bands not well delimited. Ventral fur long (LVF ~ 9.5 mm) and bicolored, slightly lighter than dorsal fur, with Brownish Olive bases that extend to about half the length of hairs, and Light Yellowish Olive tips; contrast between bands visible, but not well delimited. Wing membranes naked, dark brown. Plagiopatagium attached to the base of the toe. Dorsal surface of the uropatagium slightly paler than wing membranes, almost naked. Ventral surface of the uropatagium dark brown, with scarce hairs close to the base of the tail. Ears greatly enlarged, slightly triangular, connected by a low band of skin; tragus wider at the base, slightly curved outward, long (~ 13 mm), notched, and pointed. Muzzle broad and slightly inflated.

**Table 2.** External and skull measurements of *Histiotusalienus*. Acronyms and descriptions of the measurements are available in Table 1.

Measurement	BM 9.11.19.1 (female), holotype	MN 91624 (male)
FL	44.5	43.4
EL	27.5	27.2
WMLE	4.6	–
MAL	12.1	11.9
MAN	6.9	7.1
COH	4.6	–
GLS	18.3	16.9
CCL	16.0	14.9
CIL	17.0	–
BAL	15.2	–
ZYG	11.2	10.3
MAB	9.1	8.9
BCB	8.3	8.9
POB	4.5	4.3
BAC	5.0	4.5
BAM	7.1	6.1
MTL	6.4	5.8
M1M3	4.1	3.8
WFH	4.1	–

**Figure 4.**
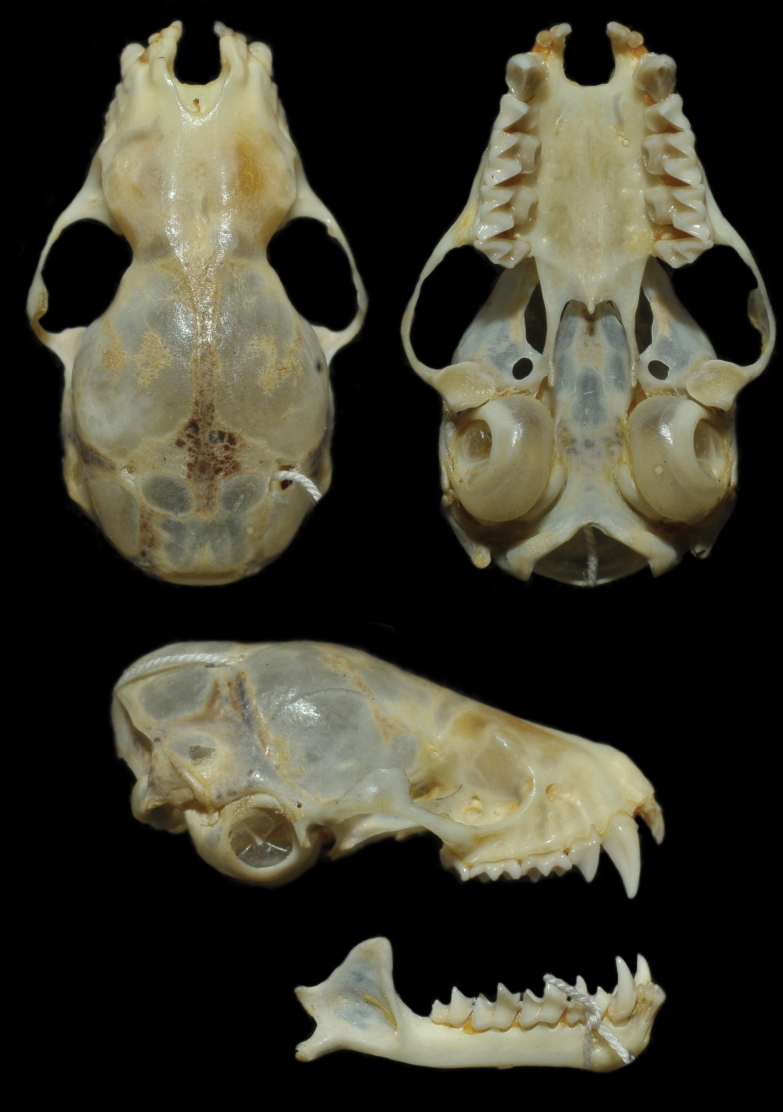
Dorsal, ventral, and lateral views of the skull of the holotype of *Histiotusalienus* Thomas, 1916 (BM 9.11.19.1).

Skull delicate; rostrum short and flattened dorsoventrally, straight in lateral profile; braincase slightly wider than the rostrum. Posterior region of the braincase rounded, regular. Nasal opening U-shaped in dorsal view. Frontals expanded laterally towards the orbit. Sagittal and lambdoidal crests weakly developed, not connected, occipital helmet absent. Triangular, flattened bony plate weakly developed where the sagittal and lambdoidal crests connect. Zygomatic arches thin and greatly widened medially. Basisphenoid pits absent. Palate extends well beyond molars, ending in a concave posterior edge, with a weakly developed medial spine (Fig. 4).

Dental formula I 2/3, C 1/1, P 1/2, M 3/3 (×2) = 32. I^1^ separated, spatulate, and strongly bilobed; wide and short, with well-developed inner and outer cusps. I^1^ about three times the size of I^2^. I^1^not aligned to I^2^ on a transversal axis of the skull. I^2^ and C^1^ separated by a small gap, C^1^ with two slightly concave faces on the lingual region, and one slightly concave face on the labial region. P^1^ well developed, reaching half of C^1^ in height; P^1^ in contact with C^1^ and molars. M^1^ and M^2^ about the same size, almost square shaped, with W-shaped cusps. M^3^ reduced, triangular, with only 3 cusps. I_1_–I_3_ reduced, trilobed, and occupying the whole space between canines. P_2_ about three times P_1_ in height. Molars have well-developed cusps and decrease in size from M_1_ to M_3_.

##### Comparisons.

*Histiotusalienus* most resembles *H.colombiae* Thomas, 1916, *H.magellanicus* (Philippi, 1866), and *H.velatus* (I. Geoffroy St.-Hilaire, 1824), from which it can be differentiated based on a series of characters. From *H.colombiae*, *H.alienus* differs in the size of the ears (>30 mm in *H.colombiae* and ~ 27 mm in *H.alienus*), development of the membrane between pinnae (poorly developed in *H.colombiae* and about 2 mm high at the edges and vestigial at the center in *H.alienus*), and length of the dorsal fur (>13 mm in *H.colombiae* and ~11.5 mm in *H.alienus*). From *H.magellanicus*, *H.alienus* differs in the size of the ears (>27 mm in *H.magellanicus*, usually close to 23 mm, and ~27 mm in *H.alienus*), width of medial lobe of ear (~3 mm in *H.magellanicus* and ~4.5 mm in *H.alienus*), shape of ears (oval in *H.magellanicus* and slightly triangular in *H.alienus*), development of the membrane between pinnae (almost absent in *H.magellanicus*, and about 2 mm high at the edges and vestigial at the center in *H.alienus*). From *H.velatus*, *H.alienus* differs in the width of medial lobe of the ears (>6 mm in *H.velatus* and ~4.5 mm in *H.alienus*), development of the membrane between pinnae (~3 mm high throughout its extent in *H.velatus*, and about 2 mm high at the edges and vestigial at the center in *H.alienus*), the shape of the ears (noticeably triangular in *H.velatus* and slightly triangular in *H.alienus*), and the length of the dorsal fur (~10 mm in *H.velatus* and ~11.5 mm in *H.alienus*). From *H.humboldti* Handley, 1996, *H.alienus* differs in the lateral profile of the skull (sharply dished in *H.humboldti* and flat in *H.alienus*), development of the membrane between pinnae (~2 mm high throughout its extent in *H.humboldti*, and about 2 mm high at the edges and vestigial at the center in *H.alienus*), and color (orangish-brown dorsal fur and light-yellowish ventral fur in *H.humboldti*, and dark-brown dorsal fur and slightly lighter ventral fur in *H.alienus*). From *H.mochica*Velazco et al., 2021, *H.alienus* can be easily differentiated by the pelage color and banding pattern (unicolored dorsal fur in *H.mochica* and bicolored in *H.alienus*), width of medial lobe of the ears (>9 mm in *H.mochica* and ~4.5 mm in *H.alienus*), development of the membrane between pinnae (~4.5 mm high throughout its extent in *H.mochica*, and about 2 mm high at the edges and vestigial at the center in *H.alienus*), shape of ears (noticeably triangular in *H.mochica* and slightly triangular in *H.alienus*). From *H.cadenai*, *H.alienus* differs in the size of the ears (>31 mm in *H.cadenai* and ~27 mm in *H.alienus*), development of the membrane between pinnae (poorly developed in *H.cadenai*, and about 2 mm high at the edges and vestigial at the center in *H.alienus*), shape of the ears (noticeably triangular in *H.cadenai* and slightly triangular in *H.alienus*), and color (yellowish general color in *H.cadenai* and dark-brown general color in *H.alienus*). From *H.diaphanopterus* Feijó, Rocha & Althoff, 2015, *H.laephotis* Thomas, 1916, *H.macrotus* (Poeppig, 1835), and *H.montanus* (Philippi & Landbeck, 1861), *H.alienus* differs in color, with general color dark brown in *H.alienus* and much lighter in the other species, and with nearly white ventral fur in *H.diaphanopterus*, *H.laephotis*, *H.macrotus*, and *H.montanus*.

##### Morphometric analysis.

**Figure 5.**
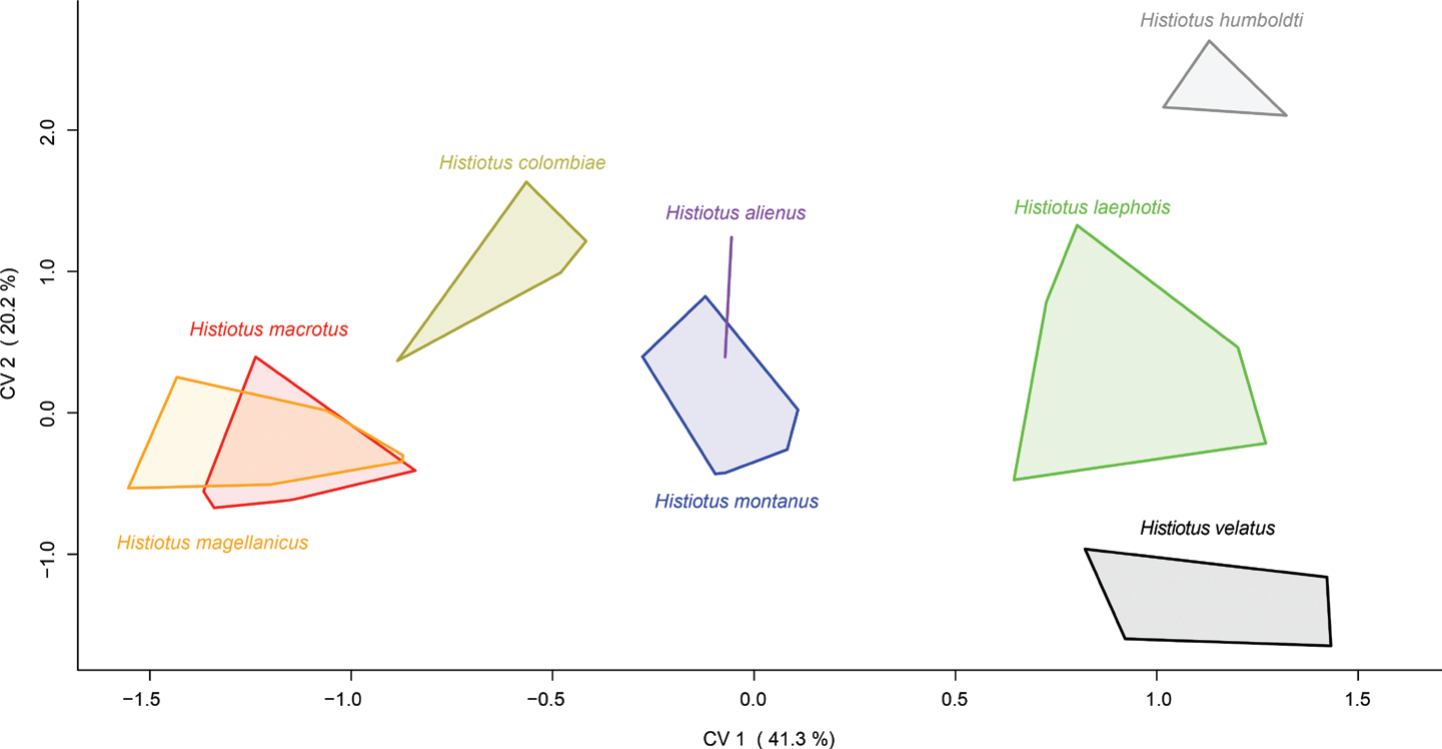
Plot of multivariate individual scores of craniometric characters in the first two canonical variates. Analyses were performed using 16 craniodental measurements.

**Figure 6.**
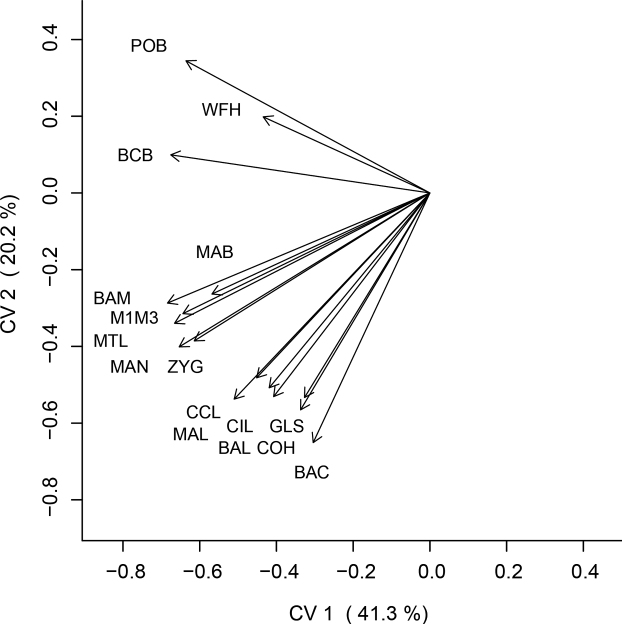
Plot of vector correlations of craniometric characters in the first two canonical variates. Analyses were performed using 16 craniodental measurements.

In the CVA, the first canonical variate CV1 accounts for 41.3% of the variation and is influenced by size, as observed in the loadings of all variables, which are all uniformly negative (Figs 5, 6). The plots along the axis of CV1 also reflect the differences in skull size among the species analyzed. *Histiotusalienus* is recovered as intermediate in size among its congeners, overlapping only with *H.montanus*. *Histiotusmagellanicus*, *H.macrotus*, and *H.colombiae* have the largest skull sizes, with *H.magellanicus* and *H.macrotus* extensively overlapping in the morphospace. The smaller *H.laephotis*, *H.humboldti*, and *H.velatus* also extensively overlap along CV1. Along CV2 (20.2% of the variation), which has a greater influence of the shape of the skull, almost all species overlap in the morphospace; this highlights the resemblance of skull shapes among *Histiotus* species. Considering the correlations of CV2 (Fig. 6), there is an evident contrast between the POB, WFH, and BCB subset of measurements with the remaining measurements, indicating some degree of differentiation in the shape of the skull between these taxa despite the overall resemblance. *Histiotusalienus* is recovered as most similar to *H.montanus* in the morphometric analysis, which considers skull shape and size; however, these species are strikingly different in their external morphology and easily distinguished.

## ﻿Discussion

The amended diagnosis of *Histiotusalienus* aims to facilitate the diagnosis of the species both in the field and in museum collections. The review of specimens, mainly from southern Brazil, already in scientific collections could reveal additional records of *H.alienus*. The general results of our morphometric analysis indicate some degree of similarity among species in the shapes of their skulls, which demonstrates the usefulness of external morphology in correctly identifying *Histiotus* species.

We reinforce the need for a broad taxonomic review of *Histiotus* and suggest that other species not yet described likely exist, as attested by recent studies (Rodríguez-Posada et al. 2021; Velazco et al. 2021). Most descriptions of *Histiotus* species are more than a century old and somewhat vague, and the taxonomic limits between species are not clear. Here, we hope to aid in the correct identification and delimitation of *Histiotus* species.

The lack of information on the natural history of *H.alienus* and its apparent rarity, with only two records in over more than 100 years, has led to its classification as Data Deficient by the International Union for the Conservation of Nature (González and Barquez 2016; Cláudio 2019). This species is associated with the Atlantic Forest, which is highly fragmented due to historical land occupation and is currently under pressure from agricultural activities (Ribeiro et al. 2009). In Palmas, threats to grasslands include the growth of the wind power sector and interests of the hydroelectric energy sector in the Chopim river basin. Despite that, the new record of *H.alienus* in Palmas is in a protected area, which indicates that at least one population of the species may be protected. We note the importance of protected areas for the maintenance of wildlife such as this species.

## Supplementary Material

XML Treatment for Histiotus alienus

## ﻿Additional information

### Author ORCIDs

Vinícius C. Cláudio https://orcid.org/0000-0002-3438-911X

Brunna Almeida https://orcid.org/0000-0003-1225-0683

Roberto L. M. Novaes https://orcid.org/0000-0003-1657-2807

Marcos A. Navarro https://orcid.org/0009-0002-5348-0987

Liliani M. Tiepolo https://orcid.org/0000-0002-4488-2768

Ricardo Moratelli https://orcid.org/0000-0003-0942-6633

### Data availability

All of the data that support the findings of this study are available in the main text.

## ﻿Appendix I

List of specimens included in morphological and morphometric analyses. Voucher material consists of stuffed skins, fluid preserved specimens, and skulls deposited in the collections (see Materials and Methods). Localities are arranged alphabetically by species and major political units. Specimens marked with asterisks were included in the canonical variate analysis.

*Histiotusalienus*.—BRAZIL (*N* = 2): Paraná, Palmas, Refúgio de Vida Silvestre dos Campos de Palmas (MN 91624*); Santa Catarina, Joinville (BM 9.11.19.1*, holotype of *Histiotusalienus*).

*Histiotuscolombiae*.—COLOMBIA (*N* = 11): Cundinamarca, Bogotá (FMNH 72165*, 72166*, 72167*, 72168*, 72169, 72170*, 72171*, 72172–72174); Cundinamarca, Coachi (BM 99.11.4.1).

*Histiotusdiaphanopterus*.—BRAZIL (*N* = 1): Maranhão, Tranqueira (FMNH 26466); BOLIVIA (*N* = 1): Santa Cruz, 5.5 km NNW of Valle Grande (AMNH 264086).

*Histiotushumboldti*.—VENEZUELA (*N* = 4): Amazonas, Cerro Neblina, 2.8 km NE Pico Phelps (USNM 560627*); Distrito Federal, Caracas, 5 mi N of Caracas (USNM 370967*); Distrito Federal, Caracas, 9.4 km N of Caracas, Hotel Humboldti (USNM 370970*); Mérida (MNHN-ZM-MO 1972-762*).

*Histiotuslaephotis*.—ARGENTINA (*N* = 12): Catamarca, Cuesta del Clavillo (CML 5253*); Catamarca, Paclin (CML 10833*); Jujuy, Cueva del Tigre (MACN 16811); Jujuy, San Pedro (CML 7058*); Salta, La Vina, Iglesia (MACN 16810); Salta, Río das Piedras (BM 34.11.4.1, 34.11.4.2); Tucumán, Tucumán (BM 2.1.5.1*, 4.10.2.1*); Tucumán, Burruyacú, El Naranjo (MACN 16814); Tucumán, Horco Molle (CML 4515*); Tucumán, Yerba Buena (CML 6103*). BOLIVIA (*N* = 7): Cochabamba, Pocana (BM 34.9.2.20); Pilcomayo, San Francisco Misiones (BM 97.2.25.3); Caiza (BM 97.2.25.1*, 97.2.25.2, 97.2.25.4*); Locality unknown (BM 45.11.18.1, 45.11.18.2). PERU (*N* = 2): Cuzco, Huasampilla (BM 73.7.3.4); Huancavelica (locality unknown, BM 38.9.26.3*).

*Histiotusmacrotus*.—ARGENTINA (*N* = 6): Catamarca, 5 km NW Chumbicha (CML 7894); Catamarca, Dique el Potrero (CML 6061); Neuquén, Parque Nacional Nahuel Huapi (CML 9884); Salta, 20 km N Cafayate (CML 5406); Tucumán, Chicligasta (CML 6185); Tucumán, Pueblo Viejo (CML 6059). CHILE (*N* = 13): Santiago (BM 35.11.10.1*, 35.11.10.3*, 35.11.10.4, 35.11.10.5*, 35.11.10.6*, 35.11.10.9, 35.11.10.13*, 35.11.10.14*, 35.11.10.16*, 35.11.10.17*, 35.11.10.18, 35.11.10.19); Locality unknown (MNHN-ZM-MO 1999-962). PERU (*N* = 2): Ancash, Huari, 1 Km W of Picheu, mouth of coal mine (FMNH 129207); Huanuco, E slope Cordillera Carpish, Carretera Central (LSU 12587).

*Histiotusmagellanicus*.—ARGENTINA (N = 8): Chubut, Río Turbio (MACN 16505); Neuquén (CML 3231); Neuquén, Los Lagos (CML 10853, 10854); Neuquén, Parque Nacional Nahuel Huapi (CML 9887); Río Negro, Bariloche (MACN 23650); Río Negro, El Bolson (LSU 16784); Tierra del Fuego, Viamonte (BM 30.10.9.1). CHILE (*N* = 12): Aisen, Almirante Simpson, Isla Gran Guaiteca (FMNH 127477*, 127478*, 127479*, 127480*); La Araucania, Cautin, Lake Gualletue (FMNH 23624*); La Araucania, Malleco, Curacautín (FMNH 23622, 23623*); Los Lagos, Chiloé, Río Inio (FMNH 23619*, 23620*); Los Lagos, Valdivia, Mafil (FMNH 23621*); Maquehue (BM 8.3.1.1*); Patagonia, Last Hope Inlet (BM 7.4.5.1).

*Histiotusmochica*.—PERU (*N* = 1): Piura, Talara, Quebrada Pariñas, 9.6 km NE of Talara (AMNH 278521).

*Histiotusmontanus*.—ARGENTINA (*N* = 11): Catamarca, Las Estarcias (CML 1758); Chubut, Pico Salamanca (28.12.11.1*); Cordoba, El Carrizal (BM 16.1.6.1*, 16.1.6.2*); Neuquén, Catán, Las Coloradas (MACN 13844); San Luis (locality unknown, MACN 16809); San Juan, Jachal (CML 5568*); Santa Cruz, Punta Loyola (BM 20.11.29.1*); Tucumán, Burruyacú, Anta Mapu (MACN 16813); Tucumán, Burruyacú, El Naranjo (MACN 16815); Tucumán, Nareu (BM 4.10.2.2*). CHILE (*N* = 5): Santiago, Punta Alta (BM 3.7.3.2*); Locality unknown (BM 49.12.4.26, 43.12.16.15, 73.12.16.14; MNHN-ZM-MO 1874-53*). ECUADOR (*N* = 1): Pichincha, Quito (MNHN-ZM-MO 1904-1179). PERU (*N* = 6): Arequipa, Islay, Chucarapi (FMNH 50780,50781); Cuzco, ca 14 km NE Alba Malaga on Ollantaitambo-Quillabamba (LSU 19215); Huancavelica, Angaraes, Lircay (FMNH 75149); San Martín, Puerta del Monte, ca 30 km NE Los Alisos (LSU 27260); Western coast (BM 68.4.29.7). URUGUAY (*N* = 3): Riviera, Riviera (FMNH 65634, 65635); Soriano (locality unknown, BM 94.1.24.8).

*Histiotusvelatus*.—ARGENTINA (*N* = 9): Corrientes, Virasoro (MACN 18055); Jujuy, Manuel Belgrano (CML 7059, 11916); Misiones, Oberá (MACN 18053, 18054, 18055, 18056–18059). BRAZIL (*N* = 22): Maranhão, Tranqueira (FMNH 26466); Mato Grosso, Chapada (BM 3.7.7.17); Minas Gerais, Lagoa Santa (FMNH 20744; MN 6516); Minas Gerais, Viçosa (MN 3395); Rio de Janeiro, Ilha Grande (MN 23071, 23072); Rio de Janeiro, Itaguaí (MACN 16812); Rio de Janeiro, Rio de Janeiro, Quinta da Boa Vista (MN 3547, 23049); Rio de Janeiro, Seropédica (ALP 1522*, 1579*, 1581*, 2096*, 2349*, 2350*, 4845*, 4942*, 5088*, 5595*); São Paulo, Pirassununga (MN 23048); Santa Teresa (MNHN-ZM-MO 1999-963). PARAGUAY (*N* = 1): Colonia Asunción (MACN 16808). PERU (*N* = 11): Cuzco, Quispicanchi (FMNH 66389, 66391, 66393, 68496–68502, 68504, 68504).
